# Supplementary material for: Engineering an fgfr4 knockout zebrafish to study its role in development and disease
Source: PLoS One. 2024 Nov 22;19(11):e0310100. doi: 10.1371/journal.pone.0310100 (PMC11584112; doi:10.1371/journal.pone.0310100)
Supplement: S1 Table — (PDF) [file pone.0310100.s001.pdf]

Oligonucleotides	Sequence
fgfr4 gRNA	Guide 1: GAATCTTCATAGGTAACACCGA
	Guide 2: AAGAGGCTCTACGCAACTCCGA
fgfr4 HRMA/A-Tail Cloning primers	FWD: 5'-GTGGTTCAAGAGTGGTGTTC-3'
	REV: 5'-CTGCCACTGTGATAGTGAAG-3'
fgfr4 genotyping primers	FWD: 5'-CCAGTGGTTCAAGAGTGGTGTTC-3'
	REV: 5'- CTACCTGCCACTGTGATAGTGAAG-3'
fgfr4 qRT-PCR primers (5' of mutation)	FWD: 5'-AGTGGTTCAAGAAGGACAGTAA-3'
	REV: 5'-AAGGAAATATGTTGACTTTGGAGAG-3'
fgfr4 qRT-PCR primers (3' of mutation)	FWD: 5'-GTGAAGATTGTGAAGACAGGAAGC-3'
	REV: 5'-GGCTACATCCTCCTCTGATAAGAC-3'
gapdh qRT-PCR primers (Kendall et al., 2018)	FWD: 5'-GTGGCCATCAATGACCCATTC-3'
	REV: 5'-CAATGACCAGTTTGCCGCCTTC-3'
rpl13a qRT-PCR primers (Kendall et al. , 2018)	FWD: 5'-CGGTCGTCTTTCCGCTATT-3'
	REV: 5'-TTCCAGAGATGTTGATACCCTCAC-3'
