# Supplementary material for: Engineering an fgfr4 knockout zebrafish to study its role in development and disease
Source: PLoS One. 2024 Nov 22;19(11):e0310100. doi: 10.1371/journal.pone.0310100 (PMC11584112; doi:10.1371/journal.pone.0310100)
Supplement: S2 Table — Genotypes obtained from heterozygous in-crosses of each fgfr4 knockout strain. Differences between observed and expected wildtype, heterozygous, and homozygous genotype distribution were nonsignificant by chi-square statistical tests. P values for actual and expected genotypes were 0.2950, 0.2706, and 0.3917 for fgfr4nch4, fgfr4nch5, and fgfr4nch6 respectively. (PDF) [file pone.0310100.s002.pdf]

	fgfr4 nch4		*fgfr4* nch5		*fgfr4* nch6	
	Number	%	Number	%	Number	%
Homozygotes	35	17.12	38	21.69	26	28.4
Heterozygotes	57	51.35	110	58.2	32	39.5
Wildtype	19	31.53	41	20.11	23	32.1
Total	111	100	189	100	81	100
