# Supplementary material for: Engineering an fgfr4 knockout zebrafish to study its role in development and disease
Source: PLoS One. 2024 Nov 22;19(11):e0310100. doi: 10.1371/journal.pone.0310100 (PMC11584112; doi:10.1371/journal.pone.0310100)
Supplement: S3 Table — Sex split data obtained from homozygous fgfr4 mutant zebrafish colonies. Differences between male and female observed and expected sex distributions were nonsignificant by chi-square statistical tests. P values for actual and expected sexes were 0.6184, 0.7891, and 0.5623 for fgfr4nch4, fgfr4nch5, and fgfr4nch6 respectively. (PDF) [file pone.0310100.s003.pdf]

	fgfr4 ^{nch4}		*fgfr4* ^{nch5}		*fgfr4* ^{nch6}	
	Number	%	Number	%	Number	%
Female	14	58.3	28	54.9	13	46.4
Male	10	41.7	23	45.1	15	53.6
Total	24	100	51	100	28	100
