# Supplementary figures and images for: Engineering an fgfr4 knockout zebrafish to study its role in development and disease
Source: PLoS One. 2024 Nov 22;19(11):e0310100. doi: 10.1371/journal.pone.0310100 (PMC11584112; doi:10.1371/journal.pone.0310100)

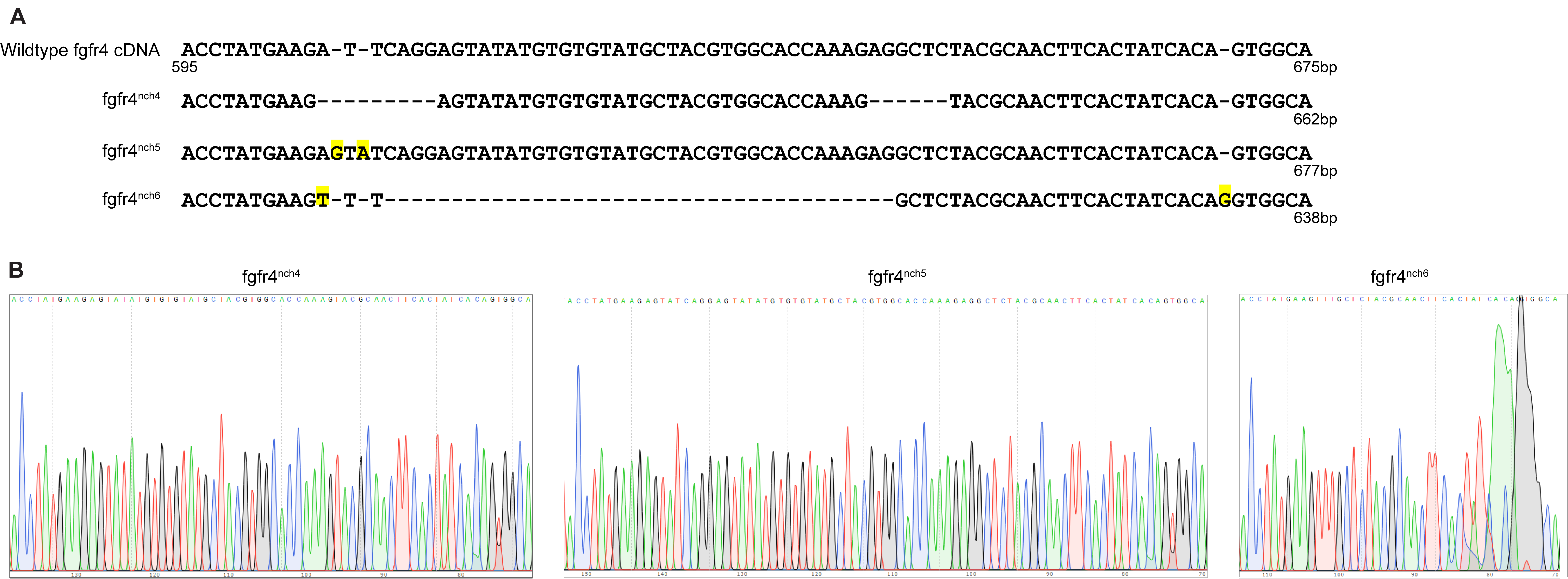

Supplement: S1 Fig — (A) Mutation sequence alignments to wildtype fgfr4 cDNA. Wildtype reference is Danio rerio fgfr4 cDNA from genome assembly GRCz11, NM_131430.1, chr21:37183912–37194363. Strain fgfr4nch4 contains a 7 base pair (bp) deletion at 604-610bp (GATTCAG/-) and 6bp deletion at 644-649bp (AGGCTC/-), strain fgfr4nch5 has two 1bp insertion after 605bp (-/G) and 606bp (-/A), and strain fgfr4nch6 has one substitution at 609bp (A/T), 38bp deletion at 609-646bp (CAGGAGTATATGTGTGTATGCTACGTGGCACCAAAGAG/-), and a 1bp insertion after 669bp (-/G). Highlighting indicates inserted and mismatched base pairs. (B) Trace sequencing files for all fgfr4 mutants. (TIF) [file pone.0310100.s004.tif]

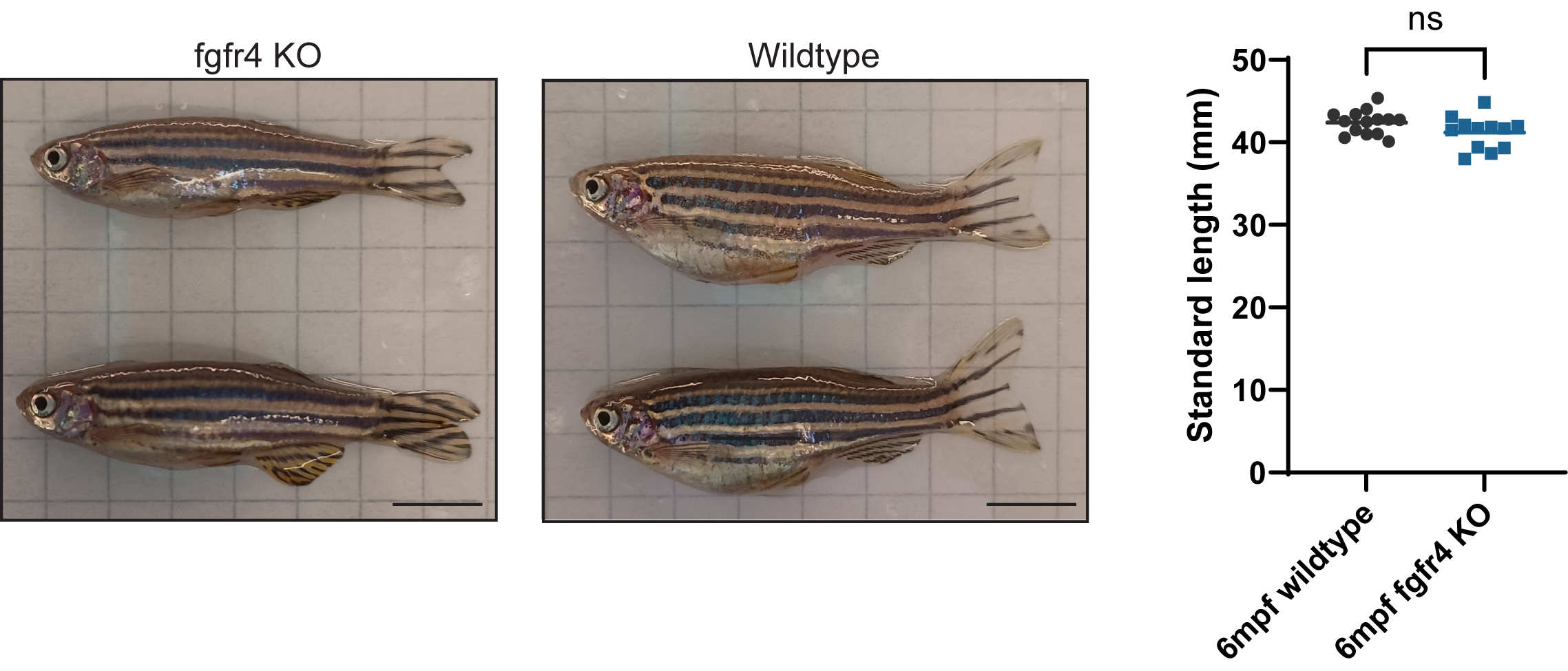

Supplement: S2 Fig — Standard length quantification was performed with n = 14 Wildtype (WT) fish and n = 12 fgfr4 KO fish. Each point represents an individual fish standard length, and the bar represents the mean. An unpaired two-tailed t-test with Welch’s correction was used to calculate the p value. Scale bar is 1 cm. (TIF) [file pone.0310100.s005.tif]
